# Electron transfer and ROS production in brain mitochondria of intertidal and subtidal triplefin fish (*Tripterygiidae*)

**DOI:** 10.1007/s00360-023-01495-4

**Published:** 2023-05-05

**Authors:** Jules B. L. Devaux, Chris P. Hedges, Nigel Birch, Neill Herbert, Gillian M. C. Renshaw, Anthony J. R. Hickey

**Affiliations:** 1 School of Biological Sciences, The University of Auckland, Auckland Mail Centre, Private Bag 92019, Auckland, 1142 New Zealand; 2 Institute of Marine Science, The University Auckland, Auckland, 1142 New Zealand; 3 School of Allied Health Sciences, Griffith University, Gold Coast Campus, Gold Coast, QLD 4222 Australia

**Keywords:** Hypoxia, Succinate, Reactive oxygen species, Hypoxia tolerance, Respirometry

## Abstract

While oxygen is essential for oxidative phosphorylation, O_2_ can form reactive species (ROS) when interacting with electrons of mitochondrial electron transport system. ROS is dependent on O_2_ pressure (PO_2_) and has traditionally been assessed in O_2_ saturated media, PO_2_ at which mitochondria do not typically function in vivo. Mitochondrial ROS can be significantly elevated by the respiratory complex II substrate succinate, which can accumulate within hypoxic tissues, and this is exacerbated further with reoxygenation. Intertidal species are repetitively exposed to extreme O_2_ fluctuations, and have likely evolved strategies to avoid excess ROS production. We evaluated mitochondrial electron leakage and ROS production in permeabilized brain of intertidal and subtidal triplefin fish species from hyperoxia to anoxia, and assessed the effect of anoxia reoxygenation and the influence of increasing succinate concentrations. At typical intracellular PO_2_, net ROS production was similar among all species; however at elevated PO_2_, brain tissues of the intertidal triplefin fish released less ROS than subtidal species. In addition, following in vitro anoxia reoxygenation, electron transfer mediated by succinate titration was better directed to respiration, and not to ROS production for intertidal species. Overall, these data indicate that intertidal triplefin fish species better manage electrons within the ETS, from hypoxic–hyperoxic transitions.

**Supplementary Information:**

The online version contains supplementary material available at 10.1007/s00360-023-01495-4.

## Introduction

Oxygen is essential to sustain life for multicellular aerobic organisms (Brahimi-Horn and Pouyssegur 2007) as it is used to efficiently release and conserve energy in phosphate bonds of ATP via oxidative phosphorylation (OxPhos) (Mitchell 1966). While over 90% of the O_2_ consumed is utilized by mitochondria for ATP production, under rare conditions where phosphorylation is impeded, up to 2% of the electrons may leak from the mitochondrial electron transport system (ETS) and rapidly react with the surrounding O_2_ to form reactive oxygen species (ROS) (Solaini et al. 2010). The latter may react directly with proteins, lipids, nucleic acids, and electrons, and can in excess compromise tissue functions if antioxidant responses and repair processes are limiting (Dickinson and Chang 2011).

While the probability of ROS formation likely increases as the mitochondrial membrane potential is elevated, and where the electron transport system is overly reduced, it is also dependent on the O_2_ concentration (Makrecka-Kuka et al. 2015; Scandurra and Gnaiger 2010). Therefore, although while O_2_ is essential for OxPhos, paradoxically it is toxic in excess. Electron leakage from mitochondria appears to result prominently at the FMN site of complex I (CI), and at complex III (CIII), where the latter interacts with the CoQ pool (Murphy 2009; Turrens et al. 1985). Perhaps of greater physiological relevance, complex II (“CII”; or succinate dehydrogenase, SDH) has been shown to mediate a process of “reverse electron transfer” (RET) on reperfusion (Chouchani et al. 2014). With ischemia, succinate elevates as the ETS slows as O_2_ availability declines. Indeed, succinate elevates several fold higher than all other tricarboxylic acid cycle intermediates in all tissues through a reversal of succinate dehydrogenase (Chouchani et al. 2014). On reperfusion, rapid oxidation of succinate at CII fully reduces coenzyme Q pools (Q to QH_2_), and this appears to promote QH_2_ flow to CI instead of CIII and mediates electron leakage to form superoxide (Chouchani et al. 2014). Historically, succinate-mediated ROS production has been explored experimentally in the presence of rotenone, a CI inhibitor (Degli 1998). The specific artificial effects of rotenone remain unclear since it can increase or decrease ROS production, dependent on species, tissue, and mitochondrial respiration state (Zorov et al. 2014).

While much attention has been placed on the role of succinate and mitochondrial ROS release in clinical contexts (Pell et al. 2016), the implications of ROS production within organs of animals that naturally and frequently are exposed to hypoxia reoxygenation and even hyperoxia warrant investigation, as these animals may have circumvented issues or can efficiently buffer mitochondria from oxidative stressors (Pell et al. 2016; Andrienko et al. 2017; Wijermars et al. 2016; Pfleger et al. 2015; Tretter et al. 2016; Valls-Lacalle et al. 2016). Relative to terrestrial species, fish are more prone to varying O_2_ levels and the associated oxidative stress (Lushchak and Bagnyukova 2006). In the intertidal zone, stagnant rock pool water can reach near anoxic levels at night, but they can also rise to 300% O_2_ saturation on sunny summer days when photosynthesis is active (McArley et al. 2018; Richards 2011). Therefore, intertidal fish are frequently exposed to both hypoxia and hyperoxia. While there are only a few studies that have explored mitochondrial ROS production in fish tissues exposed to hypoxia (Du et al. 2016; Hickey et al. 2012; Pelster et al. 2018; Zeng et al. 2016), we have found only one study exploring hyperoxic oxidative stress in fish, the demersal Atlantic cod (*Gadus* *morhua*), which was exposed to O_2_ at 200% air saturation (Karlsson et al. 2011a). Notably elevated PO_2_ also elevates mitochondrial ROS formation (Makrecka-Kuka et al. 2015), and ROS production has generally been assessed at an initial PO_2_ that is significantly higher than that likely occurs at normoxic cellular levels.

Intertidal New Zealand triplefin fish (*Tripterygiidae* family) frequently experience episodes of hypoxia reoxygenation in their ecological niches, and due to photosynthesis within pools, they also experience hyperoxia, with PO_2_ levels exceeding 200% air saturation in rock pools during sunny summer days (McArley et al. 2018). This likely increases intracellular PO_2_ and should increase the likelihood of ROS formation (Karlsson et al. 2011b). The close phylogenetic background of the 26 endemic species (Hickey and Clements 2005) makes these fish a great model to explore adaptations, such as those required to survive hypoxic, and the less often considered, hyperoxic environments (Hickey et al. 2009).

Here, we use brain, a tissue that is highly dependent on aerobic metabolism, to compare mitochondrial respiration and ROS production of three intertidal and hypoxia-tolerant species (HTS) to a hypoxia-sensitive subtidal species. Habitat distribution of the four species is presented in Figure S1. We tested how ROS production changes with gradual accumulation/titration of succinate, a substrate that accumulates in hypoxia in vertebrates (Chouchani et al. 2014), and we tested the effects of anoxia reoxygenation in vitro and ROS production in the contexts of O_2_ concentration, from a relative hyperoxia to anoxia.

## Materials and methods

### Animal sampling and housing

Four triplefin fish species with various degrees of hypoxia tolerance (Hickey et al. 2009; McArley et al. 2019) were chosen for this study. The exclusively intertidal Bellapiscis medius was the most HTS and was caught in exposed rock pools at low tide. Two “intermediate” species *Forsterygion* *lapillum* and *F.* *capito*, occupy intertidal waters and can be found in low rock pools and sub-tidally (> 5 m) and were caught in exposed rock pools at low tide and off estuarine piers. The most hypoxia-sensitive *F.* *varium* occupies subtidal waters and was caught while scuba. Adult specimens were housed in flow through aerated sea water (20 °C) and fed with mixture of shrimp and green lipped mussels for at least 2 weeks. Animal capture, handling, and experimental procedures were approved by the University of Auckland Ethics Committee (R001551).

### Brain sampling and permeabilization process

Fish were euthanized by sectioning the spinal cord and the whole brain was quickly dissected and placed in ice-cold preservation media (in mM: 2.77 CaK_2_EGTA, 7.23 K_2_EGTA, 5.77 Na_2_ATP, 6.56 MgCl_2_.6H_2_O, 20 taurine, 15 Na_2_ phosphocreatine, 20 imidazole, 0.5 DTT, 50 KMES, and 30 sucrose, pH 7.24 at 20 °C). The whole brain (~ 12 mg) was partitioned into small sections of ~ 4 mm^2^ and cellular permeabilization was undertaken with the addition of 50 µg ml^−1^ freshly prepared saponin and gently shaken for 30 min at 4 °C. The brain sections were then transferred into three subsequent ice-cold respiration medium baths (0.5 EGTA, 3 MgCl_2_.6H_2_O, 60 K-lactobionate, 20 taurine, 10 KH_2_PO_4_, 2.5 HEPES, 30 MES, 160 sucrose, 1 g l^−1^ BSA, pH 7.24 at 20 °C) and mixed gently at 4 °C for 10 min. Sections of brain were then blotted dried and weighed prior to use for respiration assays. Randomized sections selection was prioritized over selection of brain regions to grasp the overall brain mitochondrial response of the whole brain and cellular populations. Permeabilization was chosen over other tissue preparations (e.g., homogenization and mitochondrial isolation) as it avoids excess shear stress and better retains mitochondrial integrity in situ and allows direct measurement of mitochondrial responses without interference from cellular regulations, which is a critical aspect of this study.

### Respirometry and ROS assays

O2k Oroboros™ oxygraphs (Innsbruck, Austria) were used for the simultaneous measurement of mitochondrial respiration and ROS production was quantified using the Amplex UltraRed™ -horseradish peroxidase as described elsewhere (Hickey et al. 2012). Oxygen sensors were calibrated prior to each experimental procedure to account for the instrumental and chemical background flux and this includes time response of the electrode near anoxia. Around 5 mg of freshly permeabilized triplefin brain was introduced into the respirometry chambers containing respiration medium at 20 °C (261.9 µM O_2_ = 20.5 kPa PO_2_ at 101.5 kPa barometric pressure). Assays consisted of the sequential addition of substrates, inhibitors, and uncoupler to test the mitochondrial respiration, ROS production, and electron leakage under various conditions, detailed in Table 1. These conditions included (i) at decreasing PO_2_, (ii) post anoxia reoxygenation in the chamber, (iii) mediated by either complex I or II (CI and CII, respectively) in the absence or presence of rotenone, and (iv) mediated by graded succinate. Experiments were designed so the applied condition and control were run on samples from the same individual. O_2_ fluxes attributed to oxidative phosphorylation (OxPhos) were measured in the presence of saturated: pyruvate (10 mM), malate (5 mM), glutamate (10 mM), succinate (10 mM), and ADP (1 mM). Samples were left to deplete O_2_ until anoxia was reached, held for 20 min and then reoxygenated (up to ~ 240 µM O_2_ = 18.8 kPa PO_2_). Control groups were run in a parallel chamber and held with sufficient O_2_ for the same length of time. Respiration that was not attributable to OxPhos, i.e., proton leak (Leak) was measured with the addition of the ATP_F0-F1_ synthase inhibitor oligomycin (2.5 µM) and the adenylate translocase inhibitor carboxyatractyloside (0.75 mM). Potassium cyanide (1 mM) was then added to account for residual O_2_ consumption.

**Table 1:** Respirometry assays employed to assess the effect of anoxia reoxygenation, graded succinate and complex contribution on the mitochondrial respiration and ROS production in permeabilized brain of New Zealand triplefin fish species

State	Assay 1 (Figs. 2, 3)		Assay 2 (Fig. 4)			Assay 3 (Fig. 5)	
	Control	Experimental			+ Rotenone	Control	Experimental
Leak	PMG + succinate	PMG + succinate	PMG	Succinate	Succinate	PMG	PMG
OxPhos	ADP	ADP	ADP	ADP	ADP	ADP	ADP
		20-min anoxia	Succinate	PMG	PMG		20-min anoxia
		Reoxygenation					Reoxygenation
						Succinate titration	Succinate titration
Leak	Oligomycin c-Atractyloside	Oligomycin c-Atractyloside				Oligomycin c-Atractyloside	Oligomycin c-Atractyloside
Uncoupled	CCCP	CCCP				CCCP	CCCP
Resting O_2_ consumption	KCN	KCN	KCN	KCN	KCN	KCN	KCN

Freshly permeabilized brain was partitioned equally between conditions for each assay chambers, containing fully aerated respiration medium thermostated at 20ºC. Mitochondrial respiration and ROS production were measured simultaneously using Oroboros™ O2ks

*P* pyruvate, *G* glutamate, *ADP* adenosine diphosphate, *CCCP* carbonyl cyanide m-chlorophenyl hydrazone, *KCN* potassium cyanide

The maximum contribution of CI and CII to respiration, ROS production, and electron leakage was assessed in multiple settings. First, with saturating CI substrates (pyruvate, malate, and glutamate) + ADP. The additive effects of CII (“+CII”) was then measured following the addition of saturating succinate. Net CII contribution to OxPhos was then assessed in separated assays with saturating succinate + ADP, with or without the CI inhibitor rotenone (0.5 µM), and additional CI (“+CI”) was then mediated by the addition of the CI substrates stated above.

The contributions of accumulating succinate to ROS production were also measured in different settings. Succinate was first titrated in the absence of additional CI substrates and ADP (to mimic ischemic depletion of adenylates). Respiration and ROS production were then also measured in the presence of saturating CI substrates and ADP. In this setting, succinate was titrated (0–10 mM) in samples exposed to an episode of anoxia reoxygenation (AR), while controls were held with sufficient O_2_ (such as described above) to simulate and explore the effects of anoxia reoxygenation.

### Data and statistical analysis

As ROS production is dependent on O_2_ availability (Makrecka-Kuka et al. 2015; Scandurra and Gnaiger 2010), the dependence of ROS for O_2_ at Leak and OxPhos states was calculated for each sample (Fig. S2). Linear correlations between ROS production and PO_2_ were not apparent between 5 and 25 kPa, with no difference detected among species (*P* > 0.53) and there was also no effect of anoxia reperfusion (AR, *P* > 0.40). There was, however, a stronger effect in Leak state (ROS = 0.02 ± 0.006 PO_2_ + 2.71 ± 0.98) relative to the OxPhos state (ROS = 0.012 ± 0.005 PO_2_ + 1.38 ± 0.75; *F*_1,5_ = 60; *P* < 0.001). Therefore, ROS data were corrected for PO_2_ under defined states (i.e., Leak or OxPhos) (Figs. 3B, 4B, 5B).

ROS production can also be expressed as the portion of electrons not directed to respiration, i.e., a loss of efficiency to convert the electrical gradient to proton pumping. We, therefore, calculated the relative portion of electron leakage by normalizing the ROS production by the respiration rate.

For succinate titration assays, agonist dose response (three parameters) curves were fitted (least squares method) and maximum respiration mediated by succinate and mitochondrial affinity to succinate (_app_K_M,Suc_) was extracted and compared (extra sum-of-squares F test) using GraphPad Prism. The catalytic rate (K_cat,S_) was calculated as maximum respiration divided by _app_K_MSuc_.

Respiration rates and ROS production were recorded from the calibrated signal in DatLab v7.1 and processed in Excel, and curves fitted using GraphPad Prism 7. Three-way ANOVA was first employed to assess the interaction effect of AR, succinate, and species. Since no clear interaction was found, two-way ANOVA repeated measures was employed followed by Turkey’s post hoc tests to assess (1) the effect of PO_2_ and (2) graded succinate on the mitochondrial respiration, and test for differences among species in ROS production and electron leakage. Two-way ANOVAs followed by Turkey’s post hoc tests were performed to test for the effect of AR and test for the respiratory complex contribution on ROS production and electron leakage between species. Non-linear models fitted with the least squared method were compared to find the best fit, and extracted parameters were compared among species using the extra-sum-of-squares *F* test. Significant differences were determined at *P* < 0.05. All data are presented as mean ± s.e.m of six individuals of each of the four species.

## Results

### The effect of the oxygen tension on the mitochondrial function

O_2_ flux and ROS production were measured in samples as they depleted O_2_ to estimate whether PO_2_ influenced either flux. At air saturation, the three intertidal species had greater respiration rates than the subtidal species *F.* *varium* (Fig. 2A, *P* < 0.001). As O_2_ was depleted below 12 kPa, O_2_ flux of the two “mid-tidal” species matched those of *F.* *varium*, with *B.* *medius* sustaining higher O_2_ flux at lower PO_2_ (< 2.05 kPa).

The ROS dependence on O_2_ from 20.5 to 2.1 kPa PO_2_ fitted a linear regression (Fig. 2B, *F*_1,543_ = 0.196, *P* < 0.05). In this range, the ROS production was greater in *F.* *varium* than the other three species (*F*_6,22_ = 32.82, *P* < 0.0001). However, at normoxic PO_2_ at which mitochondria most likely function in vivo (i.e., PO_2_ < 2.05 kPa), the ROS dependence on PO_2_ fitted three-parameter dose–response curves (*P* < 0.005) and these curves did not differ among species (*P* = 0.48). While the portion of electrons directed to ROS production (i.e., ROS/respiration) was similar and stable among species until 5% air saturation (Fig. 2C), on approaching anoxia (PO_2_ < 0.02 kPa) electron leakage in *F.* *varium* increased ~ fivefold, and was higher than for the intertidal species (*P* < 0.001).

### Anoxia reoxygenation impacts mitochondrial function regardless of hypoxia tolerance

The subtidal species *F.* *varium* had the lowest OxPhos flux at PO_2_ > 15.4 kPa (*P* < 0.05). However, no difference in respiration was apparent among species below that PO_2_ level (Fig. 3A). Following AR, OxPhos respiration decreased for the other species and matched this in *F.* *varium* such that they were similar among species. No differences among species were observed for Leak state flux (Fig. 3B).

In Leak and OxPhos states, ROS production was assessed at various PO_2_ in control groups and groups exposed to AR. We, therefore, could account for the influence of altered PO_2_ on ROS production (Fig. S1), and ROS production was higher from mitochondria in the Leak state than mitochondria in the OxPhos state (Fig. 3B; *F*_1,5_ = 60; *P* < 0.001). In control-non-AR samples, ROS production in OxPhos state was ~ 2 times higher in *F.* *varium* than the other species; however, no significant differences were apparent among species in the Leak state. Exposure to AR in vitro did not affect ROS production in the OxPhos state; however, ROS production decreased in the Leak state for all species relative to that prior to AR (main effect of *F*_3,15_ = 1.2, *P* = 0.02).

Electron leakage was similar among species in the presence of sufficient elevated O_2_ (i.e., PO_2_ > 2.04 kPa; Figs. 2C, 3C). However, electron leakage significantly increased approaching anoxia in *F.* *varium* only and was significantly greater than the other species at 0.01 kPa (Fig. 2C). Following AR, electron leakage decreased in *F.* *lapillum* only (*P* < 0.001) and was lower than the other species (*P* < 0.05; Fig. 3C).

### Mitochondrial complexes contribution to electron flow

We assessed the contribution of either CI or CII linked pathways and their respective additive effects (“+PMG” and “+S”) to respiration, ROS production, and to electron leakage (Fig. 4). With CI substrates (pyruvate, malate, glutamate, “PMG”) and ADP (Fig. 4 left panels), *F.* *varium* had the lowest respiration and the highest ROS production (*P* < 0.04). While the addition of saturating succinate (+S) mediated an increase in respiration in all species (*P* < 0.001), it did not affect the ROS production, which remained highest in *F.* *varium*.

In experiments commencing with saturating succinate and ADP (Fig. 4 central panels), respiration was similar across species. However, ROS production was highest in *F.* *lapillum* and *F.* *capito* relative to *B.* *medius* and *F.* *varium* (*P* < 0.01). The subsequent addition of CI substrates (“S + PMG”) mediated a significant increase in respiration in all species (*P* < 0.001), and enhanced electron leakage in *F.* *varium* only (*P* < 0.001). ROS production remained the highest in *F.* *lapillum* and was the lowest in *B.* *medius* (*P* = 0.01).

We note that succinate only (i.e., without CI substrates) mediated higher electron leakage and ROS production than when respiration was already partially fuelled by PMG (*P* < 0.05). The comparisons among pathways and their additive effects to ROS production revealed that PMG contributed equally to respiration regardless of whether it was fed prior or post-succinate. However, succinate-mediated respiration was lower when succinate was added post-PMG in all species of fish (*P* < 0.05; Fig. 3a).

With rotenone (Fig. 4 right panels), respiration rates fuelled by succinate were halved relative to without rotenone (*P* < 0.001), yet they remained the highest in *F.* *varium* relative to the other species (*P* < 0.001). Electron leakage was 75% higher in *F.* *lapillum* relative to *F.* *varium* (Fig. 4c; *P* < 0.05). While addition of PMG substrates did not affect respiration, they increased electron leakage and ROS production in all fish species (*P* < 0.01), which were twofold higher in *F.* *lapillum* relative to *B.* *medius* and *F.* *varium*.

### Succinate dose-dependent response

Mitochondrial respiration and ROS production were measured with increasing succinate concentrations (Fig. 5). Overall, mitochondrial respiration was the lowest at Leak state (Fig. 5a and Table 2; *P* < 0.001) and there were no differences among species (Table 2). In the OxPhos state, the three HTS had higher succinate-supported respiration rates (Fig. 5a; *P* < 0.05) and higher affinities (i.e., lower aK_M,S_) for succinate relative to *F.* *varium* (Table 2; *P* < 0.001). Exposure to AR significantly decreased OxPhos O_2_ flux in the three HTS (*P* < 0.001), and two-way ANOVA revealed interactions with succinate concentration (*F*_63,315_ = 3.39; *P* < 0.001). While exposure to AR did not affect maximum respiration nor aK_M,S_ in *F.* *varium*, it decreased the aK_M,S_ and O_2_ flux in the three HTS (*P* < 0.001). AR also doubled K_cat,S_ in all species, with *F.* *varium* maintaining the highest catalytic rate (10.3 s^−1^ mg^−1^ as opposed to an average of ~ 2.5 s^−1^ mg^−1^ in HTS; *P* < 0.001; Table 2).

**Table 2:** The mitochondrial affinity for succinate is lower in the hypoxia-tolerant species

Species	Leak	Maximum respiration (pmol O_2_ s^−1^ mg^−1^)		Leak	aK_M,S_ (mM succinate)		Leak	K_cat,S_ (s^−1^ mg^−1^)	
		OxPhos	OxPhos post-AR		OxPhos	OxPhos post-AR		OxPhos	OxPhos post-AR
*B.* *medius*	1.7 ± 0.3^#^	8.9 ± 3.2	6.4 ± 1.0	2.0 ± 0.9^#^	13.5 ± 7.7	3.4 ± 1.4^#^	1.2 ± 0.1	1.0 ± 0.2	2.2 ± 0.7^#^
*F.* *lapillum*	1.9 ± 0.1^#^	5.0 ± 1.5	7.5 ± 0.9	1.4 ± 0.3^#^	5.8 ± 4.0	4.4 ± 1.4	1.7 ± 0.8	1.2 ± 0.5	2.0 ± 0.5^#^
*F.* *capito*	1.8 ± 0.2^#^	10.0 ± 3.5	8.0 ± 0.9	1.6 ± 0.4^#^	11.6 ± 6.8	3.4 ± 1.0^#^	1.2 ± 0.3	1.5 ± 0.5	3.0 ± 0.8^#^
*F.* *varium*	2.0 ± 0.1	2.8 ± 0.6*	2.9 ± 0.4*	1.3 ± 0.2	1.1 ± 1.1*	0.5 ± 0.3*	1.3 ± 0.2	4.1 ± 1.4*	10.3 ± 4.0*^#^

Succinate was titrated on permeabilized brain of the four triplefin fish species in the absence of other substrates (Leak), in the presence of mitochondrial substrates (OxPhos) and brain exposed to an event of anoxia reoxygenation (OxPhos post-AR). Agonist vs. response (three parameters) curves were fitted (least squares method) and maximum respiration mediated by succinate and succinate affinity (aK_M,S_) was extracted and compared (extra sum-of-squares F test) using GraphPad Prism. The catalytic efficiency (K_cat,S_) was calculated as maximum respiration divided by aK_M,S_. One-way ANOVAs were used to test for statistical differences, chosen at *P* < 0.05 and displayed as “*” for the difference between species and as “#” for the difference between states

Succinate-mediated ROS production (Fig. 5b) and electron leak (Fig. 5c) were greatest in the Leak state for all species (*P* < 0.001). While in OxPhos, succinate mediated a slight increase in ROS production associated with an increase in electron leakage in *F.* *varium*, succinate decreased ROS production in *B.* *medius*. Above 0.5 mM succinate, ROS production remained unchanged in all species. AR did not affect the specific ROS production nor the electron leak, and independently of succinate concentration (Fig. 5b, c).

## Discussion

As with other aerobic tissues, mitochondria within brain are the main O_2_ consumers and likely are significant sources of ROS, in particular when accumulated succinate is oxidized following anoxia. Much attention has focused on mitochondrial function in mammalian models in hypoxia or following anoxia, yet mitochondria of other vertebrates, such as fish, has been much less well explored. Moreover, interpretations of ROS production are often criticized from the perspective that most cells normally exist and function at low PO_2_. Yet most experiments are conducted at elevated PO_2_ where ROS production is elevated and likely overestimates that in in vivo (Makrecka-Kuka et al. 2015). This current study found that below 2.05 kPa (i.e., in the region of a typical intracellular PO_2_), ROS production was similar among triplefin species, with higher rates of electron leakage in the HTS. However, in hyperoxia, combined CI-linked substrates and succinate oxidation mediated lower ROS production in the brain of hypoxia-tolerant triplefins in vitro, including below 2 mM succinate, a concentration that anoxic fish may encounter in vivo (Johnston and Bernard 1983). HTS also sustained higher mitochondrial respiratory capacities relative to the hypoxia-sensitive fish as they approached anoxia, and electron transfer was more efficiently directed to respiration and not to ROS production. These findings suggest that New Zealand hypoxia-tolerant fish benefit from more efficient mitochondria that may help to sustain repeated hypoxic episodes, and likely avoid oxidative damage on approaching anoxia, upon reoxygenation and in hyperoxic conditions.

### The ROS dependence on PO_2_

**Fig. 1:**
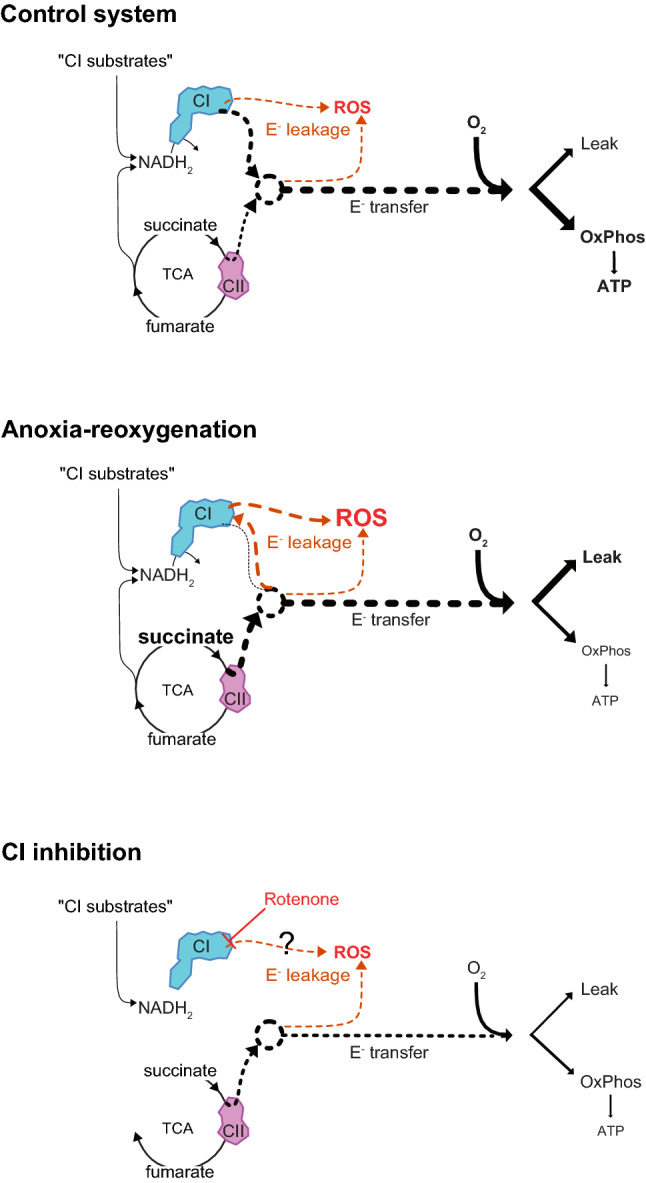
Mitochondrial electron transport system and electron transfer. With sufficient substrate and O_2_, mitochondrial substrates are metabolized to reduced equivalents, which are oxidized by either complex I (“CI”) or complex II (“CII”). As a result, electrons are stripped and directed though the electron transport system to O_2_. The free energy released from this transfer is then coupled to oxidative phosphorylation “OxPhos” and ATP production, or dissipated as proton leak, or “Leak”. Electrons may leak from the electron transfer system (ETS) and mediate the production of reactive oxygen species “ROS”. Post an anoxic event, accumulated succinate is highly reduced by CII and saturates the electron transfer at its site. This likely mediates a retro-transfer of electrons to CI that increases electron leakage and ROS production. ROS likely mediates damage to membranes, which may increase Leak. Excess ROS production may be prevented using CI inhibitor such as rotenone that blocks electron transfer at the Q-site of CI. Rotenone theoretically decreases electron leakage and associated ROS production, yet blocks the electron transfer from CI to the electron transfer system, which arrests OxPhos and ATP production. Without competition for the Q-pool, CII may have a greater contribution to the electron transfer system

As ROS production is dependent on O_2_ tension (discussed in 5), we assessed the influence of PO_2_ on ROS production and found a weak positive correlation between the ROS production and PO_2_ within the range of 4.7–22 kPa (Fig. S2). However, mitochondrial respiration state (i.e., Leak or OxPhos) influenced this O_2_ dependence. The binding kinetics of electrons to O_2_^·−^ formation are dependent on O_2_ availability and the transience of electrons in the ETS, which elevates reduction of the ETS through hyperpolarization of mitochondria. This increases this transience, and potentiates electron leakage from the ETS to form ROS (Murphy 2009; Korshunov et al. 1997; Aon et al. 2010) (Fig. 1). Here, increased electron leakage was achieved in vitro through elevating substrate and O_2_ levels, and through decreasing ADP levels (i.e., Leak state). This likely doubles the ROS production relative to the OxPhos state (Fig. S2).

**Fig. 2:**
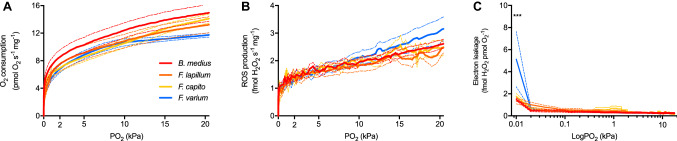
The dependence of the oxygen consumption, reactive oxygen species production, and electron leakage to decreasing oxygen tension (PO_2_). In permeabilized brain of four triplefin fish species with various degree of hypoxia tolerance, mitochondrial respiration (**A**), ROS production (**B**), and electron leakage (**C**) were assessed at decreasing PO_2_ to anoxia. Data are presented as mean ± s.e.m. of six individuals of the rock-pool species *B.* *medius* (red), the intertidal species *F.* *lapillum* (orange) and *F.* *capito* (yellow), and the subtidal species. *F.* *varium* (blue). In **C**, PO_2_ was logged to appreciate the increase in electron leakage near anoxia. Data are presented as mean ± s.e.m. (plain and dashed line, respectively). The difference between species was chosen at *P* < 0.05 or 0.001 and indicated in black as * or *** (respectively), tested with two-way ANOVA repeated measures followed by Turkey’s post hoc test

Paradoxically, hypoxia has been suggested to potentiate mitochondrial ROS production (Murphy 2009; Guzy and Schumacker 2006; Zuo et al. 2013). This phenomenon was not clear in our data and others, and this has been critically discussed elsewhere (Scandurra et al. 2010). In another study, ROS production measured with Amplex-Red™ assays, shows a linear relationship with PO_2_, in the range between 64.6 and 4.3 kPa PO_2_ (Grivennikova et al. 2018). In this present study, ROS measurements were performed in real time from ~ 20.5 to 0 kPa PO_2_ and measures were determined between 4.3 and 0 kPa PO_2_ from more than 27,500 data points (Fig. 1b), which represents the range within which mitochondria likely function within cells (Biro 2013). Within this range, the ROS–PO_2_ relationship was modeled using a three-parameter dose–response relationship (*P* < 0.02), and notably did not support a potentiation of ROS production with the onset of hypoxia. However, the portion of electrons directed toward ROS production (i.e., those not flowing to respiration) increased fourfold under 0.01 kPa PO_2_, i.e., near anoxia as respiration rates slowed (Fig. 2c). Although mitochondria are less likely to be exposed to such low oxygen tensions in most normoxic vertebrates (Benamar et al. 2008; Palacios-Callender et al. 2004), low PO_2_ is likely reached in tissues of animals exposed to periods of environmental or functional hypoxia–anoxia (Hickey et al. 2012; Bundgaard et al. 2018), or pathologically in ischemic tissues exposed for at least several minutes (Chan 2001; Bainbridge et al. 2014). We note that the amount of ROS produced by triplefin brain is small relative to other tissues, such as the heart of heat-stressed wrasses, which produced ~ 25-fold more ROS (Iftikar and Hickey 2013). To our knowledge, ROS production near anoxia has not yet been assessed in a range of species with varying tolerances to hypoxia. We conclude that the increased proportion of electron leakage near anoxia is likely due to the hyper-reduction of mitochondria, since substrate levels are high and O_2_ limiting, and this suggests that the quanta of electrons leaking from the ETS may remain fixed as O_2_ diminishes.

### Intertidal triplefins produce less ROS than the subtidal species in a saturated medium

Few studies have explored ROS production in fishes. Lower net ROS levels were observed in isolated liver mitochondria of killifish (Fundulus heteroclitus) acclimatized for a month to hypoxia or intermitted hypoxia (Du et al. 2016) and permeabilized ventricle fibers of the anoxia-tolerant epaulette shark (*Hemiscyllum* *ocellatum*) also depressed ROS release (Hickey et al. 2012). To our knowledge, only those two studies have assessed ROS production in vitro in contexts of hypoxia tolerance in fish species. This present work shows that the intertidal triplefin species produced less ROS when phosphorylating in a fully saturated medium, which is hyperoxic for mitochondria in vivo.

The three HTS of triplefin fish displayed lower CI-mediated ROS production relative to *F.* *varium*. A similar trait has been observed in liver and skeletal muscle from the mammalian hypoxia-tolerant ground squirrel, Ictidomys tridecemlineatus (Brown et al. 2012), and in heart mitochondria of the anoxia-tolerant turtle (Trachemys scripta). In the latter, decreased ROS production from CI was in part attributed to *S*-nitrosylation of specific residues within CI (Bundgaard et al. 2018).

Most investigations have been assessed in vitro at O_2_ concentrations well above those that mitochondria would likely experience in vivo. This apparent experimental artefact has been discussed by Zenteno-Savin et al. (2002) and Zenteno-Savin et al. (2002) who reported that aortic rings of diving seals, a model for ischemia–reperfusion and hypoxia tolerance, generated more ROS than tissues from pigs (Zenteno-Savin et al. 2002). In this present study, HTS generated less ROS than the hypoxia-sensitive species at high PO_2_, indicating that excess mitochondrial ROS production may be prevented in HTS triplefins. Notably, these species differ to diving seals as they occupy rock pools that can also become hyperoxic with over 200% O_2_ (McArley et al. 2018).

While the net ROS production was similar across species at lower PO_2_, and where mitochondria likely function in vivo (i.e., PO_2_ < 2.05 kPa), the fraction of ROS to O_2_ consumed, i.e., electron leakage approaching anoxia, was greatest in the hypoxia-sensitive species, indicating a tighter management of electrons in respiratory chains of the three more hypoxia-tolerant species.

### Species specific response to anoxia reoxygenation

**Fig. 3:**
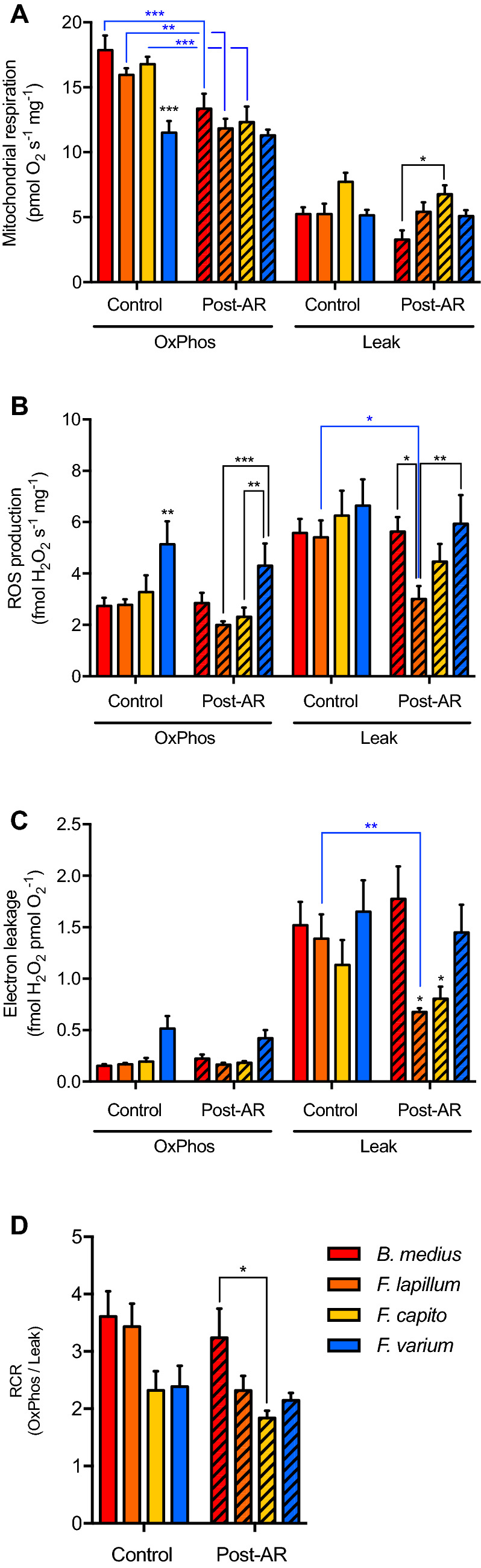
Exposure to anoxia reoxygenation (AR) in vitro. The effect of AR on the mitochondrial respiration (**A**), the ROS production (**B**), and the electron leakage (**C**) was assessed on permeabilized brain of the rock-pool species *B.* *medius* (red), the intertidal species *F.* *lapillum* (orange) and *F.* *capito* (yellow), and the subtidal species *F.* *varium* (blue). Samples were induced in OxPhos state with saturated mitochondrial substrates (pyruvate, malate, glutamate, and succinate) and ADP and let to deplete the O_2_ (~ 40 min for ~ 5 mg of brain) until anoxia. Anoxia was held for 20 min and followed by rapid reoxygenation (“post-AR”). Control groups were held with sufficient O_2_ (> 60% air saturation). Once respiration recovered and OxPhos rates post-AR measured, Leak state was induced by inhibition of the ATP_F0-F1_ with oligomycin. **D** Respiratory control ratios (RCR) were then calculated (as OxPhos /Leak) to appreciate the coupling of respiration to OxPhos. Data are presented as mean ± s.e.m. of six individuals. Statistical difference was tested with two-way ANOVA repeated measures followed by Tukey’s post-hoc test and presented in black for the difference between species, and blue for the effect of AR, as *, ** and *** for *P* < 0.05, 0.001 and 0.0001, respectively

Although absolute anoxia may not occur in rock pools (McArley et al. 2018), the PO_2_ within active tissues is likely extremely low. Therefore, we assessed the effect of 20-min anoxia in vitro. This time generally triggers mitochondria damage in other models (Rouslin 1983). On reoxygenation, OxPhos rates decreased in all HTS, and matched *F.* *varium* which respiration rates were paradoxically not affected by AR (Fig. 3b). Therefore, some damage may have occurred in HTS brain mitochondria following AR. However, Leak respiration rates were largely unaffected and the RCR remained the highest in the truly intertidal species *B.* *medius*. This indicates the greater stability of the inner mitochondrial membranes in this species, despite an overall decrease in OxPhos rates. Exposure to AR decreased the electron leakage in the intertidal species of the Forsterygion genus; however, *B.* *medius* had a similar response than *F.* *varium*. From these data, it appears that the response of the species within the Fortserygion genus follow a trend that correlates with hypoxia tolerance. However, the most HTS *B.* *medius*, species and phylogenetically more distant species (Hickey et al. 2009), had a response similar than *F.* *varium*. Therefore, in the triplefin fish species examined, the response to AR does not appear to associate with hypoxia tolerance and is species specific.

### Succinate-mediated O_2_ consumption and ROS production

**Fig. 4:**
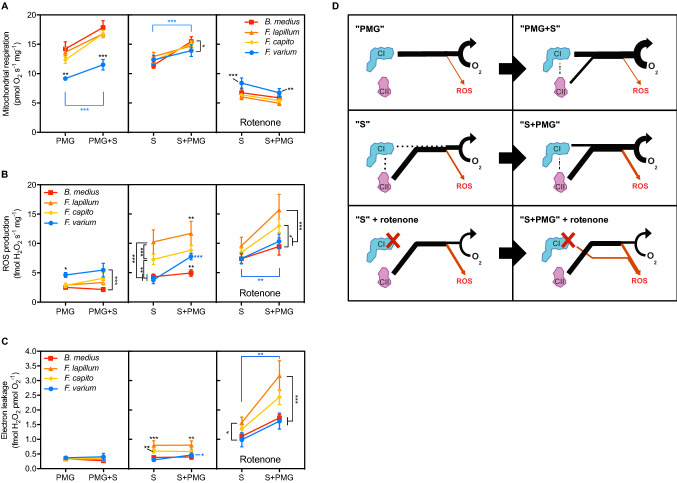
The contribution of the mitochondrial complex I (CI) and complex II (CII) to respiration and electron leakage for ROS formation. In permeabilized brain of four triplefin fish species, mitochondrial respiration (**A**) and ROS production (**B**) were measured simultaneously in three different settings. First, with ADP and CI substrates (pyruvate, malate, and glutamate; “PMG”), and subsequently, additional succinate (“PMG + S”; left panel). Second, with ADP and CII substrate (“CS”) and subsequently, additional CI substrates (“S + PMG”). Finally, the latter was repeated in the presence of the CI inhibitor rotenone. **C** The electron leakage was calculated as ROS per O_2_ consumed. **D** Graphical representation of the results. The flux of electron supported by CI or CII is represented in black. A portion of this flux not directed to respiration, electron leak (orange), is directed to ROS production. Since CII product supposedly further contributes to feeding CI, this was represented in dash. We note that the origin of electron leakage is here arbitrary. Data in **A**–**C** are presented as mean of six individuals ± s.e.m. Statistical difference was tested with two-way ANOVA repeated measures followed by Tukey’s post hoc test and is presented in black for the difference between species, and blue for the effect of the additional complex, as *, ** and *** for *P* < 0.05, 0.01 and 0.001, respectively

**Fig. 5:**
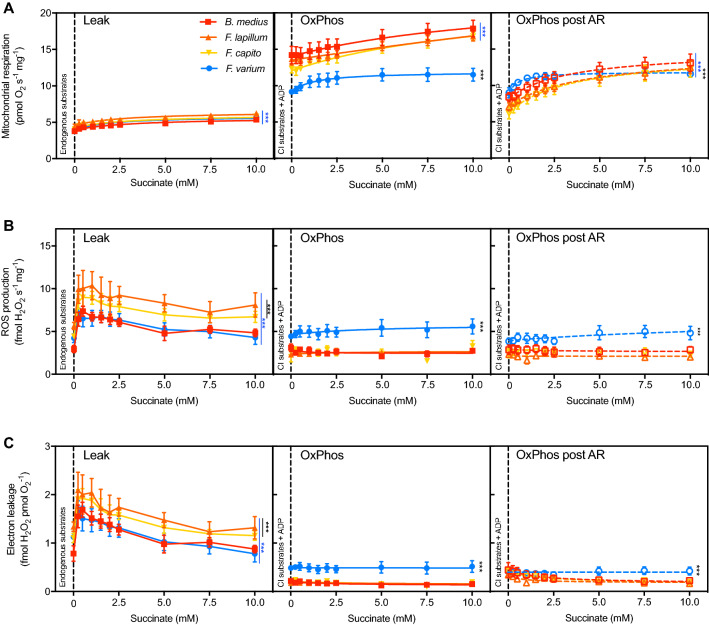
Succinate-supported respiration but not ROS production is supressed post to anoxia reoxygenation (AR) in hypoxia-tolerant triplefin fishes. Mitochondrial respiration (**A**), ROS production (**B**), and electron leakage (**C**) were assessed on permeabilized brain in Leak (endogenous substrates, no ADP nor other substrates added), OxPhos controls (additional ADP and other substrates) and OxPhos post an event of anoxia reoxygenation (“post-AR”) with graded succinate. Data are presented as the means of six individuals ± s.e.m. Warmer colors represent a greater hypoxia tolerance. Statistical difference was tested with two-way ANOVA repeated measures followed by Tukey’s post hoc test and is presented in black for the difference between species within a state, and blue for the difference between state within the same species, as *** for *P* < 0.001

In the absence of NADH-linked substrates (Fig. 4 second panel), ROS production was highest in *F.* *lapillum* and *F.* *capito* and no difference was apparent between *B.* *medius* and *F.* *varium*. However, with CI substrates present, succinate-mediated ROS was lower in the three HTS than *F.* *varium* (Fig. 5), indicating that with the more physiological CI substrates (or elevated NADH/NAD ratios, which increase in hypoxia), (Garofalo et al. 1988) ROS production is suppressed in the HTS, or is elevated in *F.* *varium*. Notably, with succinate and rotenone, ROS production was higher and electron leakage doubled in all species of fish (Fig. 4). Notably rotenone generates an artificial state, and perhaps these data illustrate the importance of mimicking substrates in vivo.

Following in vitro exposure to AR, the affinity for succinate decreased 20-fold in the 3 HTS relative to controls. However, maximal, i.e., saturating succinate oxidation rates remained unchanged. Mitochondrial “metabolic depression” mediated through suppressed succinate oxidation rates has been reported in liver and skeletal muscle of hibernating ground squirrels in metabolic suppression during torpor (Brown et al. 2013), and in hypoxic reared, or selected drosophila (Ali et al. 2012). In hypoxia-sensitive mammalian brain, high succinate oxidation rates and associated elevated ROS production have been shown to be key mediators of ischemia–reperfusion injuries (Chouchani et al. 2014). Whether lower succinate-mediated respiration rates mediated by AR were regulated or damaged associated will require further investigation. The partial suppression of succinate oxidation in vitro (Table 2) and associated diminished ROS production (Fig. 5) in hypoxia-tolerant triplefins appear to be a strategy against oxidative stress mediated by succinate overload.

## Conclusion

In this study, ROS production was assessed with the consideration of both O_2_ and substrate availability. Using simultaneous measures of respiration and ROS production, we show that the efficiency of electron transfer is greater in HTS when entering anoxia. Moreover, under hyperoxic conditions, intertidal triplefins may avoid oxidative stress with a lower ROS production relative to the subtidal species. In addition, succinate-supported respiration was more efficient for HTS and this may confer intertidal triplefins a strategy to avoid succinate-mediated oxidative stress. While paradoxically, HTS mitochondria were affected to anoxia reoxygenation, this setting is unlikely to occur in rock pools. In ecological contexts, intertidal triplefin fish better manage hypoxia and at least in vitro it appears that they likely will better manage hypoxia reoxygenation and hyperoxia, both of which occur in the unstable rock-pool environments.

## Supplementary Information

Below is the link to the electronic supplementary material. Data supporting this manuscript is available at https://doi.org/10.17608/k6.auckland.22044104.v1

Supplementary file1 (DOCX 559 KB)

## Data Availability

Data supporting this manuscript is available at 10.17608/k6.auckland.22044104.v1.

## Abbreviations

AR: Anoxia reoxygenation
CI: Mitochondrial complex I (i.e., NADH: ubiquinone oxidoreductase)
CII: Mitochondrial complex II (i.e., succinate dehydrogenase)
ETS: Mitochondrial electron transport system
HTS: Hypoxia-tolerant species
Leak: Proton leak
OxPhos: Oxidative phosphorylation
PO_2_: Partial pressure of oxygen
RCR: Respiratory control ratio
ROS: Reactive oxygen species
