# Supplementary material for: Electron transfer and ROS production in brain mitochondria of intertidal and subtidal triplefin fish (Tripterygiidae)
Source: J Comp Physiol B. 2023 May 5;193(4):413–24. doi: 10.1007/s00360-023-01495-4 (PMC10299943; doi:10.1007/s00360-023-01495-4)
Supplement: Supplementary file 1 — Supplementary file1 (DOCX 559 KB) [file 360_2023_1495_MOESM1_ESM.docx]

Supplementary data for:

Oxygen, succinate and anoxia-reoxygenation alter mitochondrial reactive oxygen species generation in the brain of intertidal fish

Authors: Jules B.L. Devaux^1^*, Chris P. Hedges^1^, Nigel Birch^1^, Neill Herbert^2^, Gillian M.C. Renshaw^3^, Anthony J.R. Hickey^1^.

^1^ School of Biological Sciences, The University of Auckland, Auckland 1142, New Zealand.

^2^ Institute of Marine Science, The University Auckland, Auckland, 1142, New Zealand.

^3^ School of Allied Health Sciences, Griffith University, Gold Coast campus, QLD 4222, Australia.

**Figure S1. Habitat distribution of the four triplefin fish species selected for this study.**

The four triplefin species selected for this study occupy the great Auckland region. *Bellapiscis medius* (red) is the only exclusive intertidal triplefin and occupies rockpools, which can become hypoxic at nocturnal low tides. *Fosterygion lapillum* (orange) and *F. capito* (yellow) are two generalist species both occasionally subjected to fluctuating environmental O_2_ levels. *F. lapillum* inhabits rockpools and shallow depths and *F. capito* is often found in estuarine and ~5m sheltered subtidal zones. *F. varium* (blue) was chosen as the exclusive subtidal species with depth of occurrence of ~8m. Triplefin images courtesy of Vivian Ward and Kendall Clements.

**Figure S2. The dependence of the ROS production to the oxygen tension**. In permeabilised brain, the ROS production was measure fluorometrically on mitochondria at OxPhos state (high substrates levels and ADP) and Leak state (inhibition of the ATP_F0-F1_ with olygomycin). Each dot represents the measurement of an individual from the rock-pool species *B. medius* (red), the intertidal species *F. lapillum* (orange) and *F. capito* (yellow) and the subtidal species *F. varium* (blue), having experienced an episode of anoxia-reoxygenation (“AR”, empty symbols) and in control groups (“Ctrl”, filled symbols). The linear correlation between ROS production and the PO_2_ at which ROS production was extracted was tested with the least squares fit and displayed with 95% confidence intervals.
